# Microbiomes and Specific Symbionts of Social Spiders: Compositional Patterns in Host Species, Populations, and Nests

**DOI:** 10.3389/fmicb.2020.01845

**Published:** 2020-07-31

**Authors:** Mette Marie Busck, Virginia Settepani, Jesper Bechsgaard, Marie Braad Lund, Trine Bilde, Andreas Schramm

**Affiliations:** ^1^Section for Microbiology, Department of Biology, Aarhus University, Aarhus, Denmark; ^2^Section for Genetics, Ecology and Evolution, Department of Biology, Aarhus University, Aarhus, Denmark

**Keywords:** symbiosis, microbiome, spider, *Stegodyphus*, *Mycoplasma*, *Borrelia*, 16S sequencing, fluorescence *in situ* hybridization

## Abstract

Social spiders have remarkably low species-wide genetic diversities, potentially increasing the relative importance of microbial symbionts for host fitness. Here we explore the bacterial microbiomes of three species of social *Stegodyphus* (*S. dumicola*, *S. mimosarum*, and *S. sarasinorum*), within and between populations, using 16S rRNA gene amplicon sequencing. The microbiomes of the three spider species were distinct but shared similarities in membership and structure. This included low overall diversity (Shannon index 0.5–1.7), strong dominance of single symbionts in individual spiders (McNaughton’s dominance index 0.68–0.93), and a core microbiome (>50% prevalence) consisting of 5–7 specific symbionts. The most abundant and prevalent symbionts were classified as Chlamydiales, *Borrelia*, and *Mycoplasma*, all representing novel, presumably *Stegodyphus*-specific lineages. *Borrelia*- and *Mycoplasma*-like symbionts were localized by fluorescence *in situ* hybridization (FISH) in the spider midgut. The microbiomes of individual spiders were highly similar within nests but often very different between nests from the same population, with only the microbiome of *S. sarasinorum* consistently reflecting host population structure. The weak population pattern in microbiome composition renders microbiome-facilitated local adaptation unlikely. However, the retention of specific symbionts across populations and species may indicate a recurrent acquisition from environmental vectors or an essential symbiotic contribution to spider phenotype.

## Introduction

Microbiomes are ubiquitous in the animal world and can provide their hosts with beneficial and sometimes essential functions like energy, nutrition, or protection against pathogens (McFall-Ngai et al., 2013). If the microbiome composition varies among host individuals and provides habitat-specific beneficial functions, microbiomes may contribute to local host adaptation (Henry et al., 2019). The composition and distribution of microbiomes among host individuals and populations may therefore provide insights into the potential of the microbiome to facilitate adaptive responses to the local environment. Depending on the transmission fidelity of the microbiome and the origin of microbiome variation, such symbiont-mediated mechanisms of host adaptation can be classified as either evolutionary (inherited), plastic (environmentally induced) or transgenerational plastic (environmentally induced and transmitted across a number of generations) (Kaltenpoth and Steiger, 2014; Zolnik et al., 2016; Näpflin and Schmid-Hempel, 2018; Rock et al., 2018; Scanlan, 2019; Vujanovic et al., 2019). A significant role in local host adaptation predicts population-specific patterns with host individuals from the same population carrying more similar microbiomes compared to host individuals from different populations. However, random forces such as low transmission fidelity and drift-like processes caused by host population dynamics may cause a less structured pattern of microbiome distribution within and across populations. The biotic and abiotic factors that shape the diversity distribution of the microbiome within individuals and populations remain poorly understood (Adair and Douglas, 2017; Reese and Dunn, 2018), although they have important implications for our understanding of the functional significance of the microbiome in host adaptation. Symbiont-mediated adaptations have been hypothesized to be especially important in species with low genetic diversity (Mueller et al., 2019), since the possibility of evolutionary responses based on standing genetic variation of such hosts is reduced (Bell, 2013; Charlesworth et al., 2017; Ørsted et al., 2019).

**FIGURE 1:**
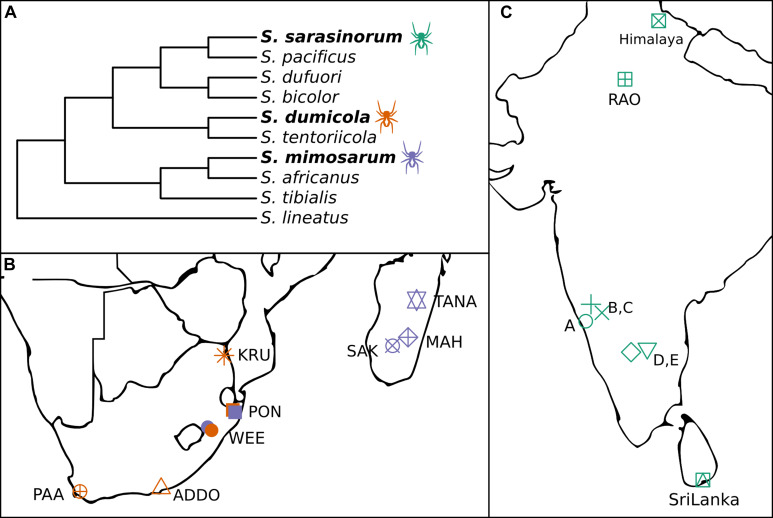
**(A)** Cladogram of the three social *Stegodyphus* (in bold) and their sub-social sister species [redrawn from Settepani et al. (2016)]. **(B,C)** Sampling locations for the three social *Stegodyphus* species across **(B)** South Africa and Madagascar, **(C)** India and Sri Lanka. Orange, *S. dumicola*; Purple, *S. mimosarum*; Green, *S. sarasinorum*.

The spider genus *Stegodyphus* (family Eresidae) contains three species (*S. sarasinorum*, *S. mimosarum*, and *S. dumicola*), that show extraordinarily low genetic diversity as consequence of an independently evolved, yet highly similar social lifestyle (Figure 1A; Johannesen et al., 2007; Settepani et al., 2016, 2017). These spiders live in nests of 100–1000 of individuals (typically 85% female) and cooperate on web building, prey capture, feeding, and brood care (Lubin and Bilde, 2007). Mothers and non-reproducing female helpers (allomothers) practice a special form of regurgitation feeding, in which they feed the spiderlings a mixture of digested prey and dissolved intestinal lining (Junghanns et al., 2019), while males die off before the next generation emerges. When the spiderlings are old enough to begin capturing prey, they consume the adult females (matriphagy), and subsequently mate and reproduce with their siblings within the nest (Lubin and Bilde, 2007). Normally, the social spiders live their entire life within their natal nest, only interacting with individuals from the same nest. Dispersal is rare, and can happen either by nest fission or by ballooning. In nest fission, one or a few females leave the nest to establish a new nest nearby, while ballooning is performed by females spinning a special silk sail which allows them to be carried long distances by the wind. In both cases, new nests are established by females who were already mated in their natal nest, allowing propagation of their family lineage with no genetic mixing (Schneider et al., 2001). Nests belonging to the same population are highly related and likely stem from a single mated ballooning female and subsequent nest-fissions (Settepani et al., 2017).

The combination of female-biased sex ratio, reproductive skew, and inbreeding (“social syndrome”) leads to enhanced genetic drift causing very low levels of genetic diversity within spider nests and populations (Settepani et al., 2017). Theoretically, this should result in drift-induced differentiation between populations, but recurrent extinction events that remove genetic variation, and recolonization events by single mated females causing strong founder effects, result in low species-wide genetic diversity (Settepani et al., 2017). Still, each spider species exists across large geographic and climatic distances (Figures 1B,C), suggesting that adaptive responses could occur by other means than based on host genetic variation, e.g., through variation in microbiome composition (Mueller et al., 2019).

In order to investigate the potential for variation in microbiome composition to assist in host adaptation in the three social *Stegodyphus* species, it is first necessary to characterize the microbiome through identification and localization of putative specific symbionts, and then to determine and quantify variation in microbiome composition within and across individuals, nests, and populations. To this end, we carried out 16S rRNA gene amplicon analysis (approx. 400 bp, V3–V4 region) on whole body DNA extractions. For each species, multiple nests were sampled from 5 to 8 geographically distinct populations (Figures 1B,C). In addition, full-length 16S rRNA gene sequences for proper phylogenetic placement of the most dominant symbionts were obtained via clone libraries, and two of the most prevalent symbionts were localized within the host by fluorescence *in situ* hybridization (FISH).

## Materials and Methods

### Sample Collection

Spiders were collected from populations spanning large climatic gradients in South Africa, Madagascar, Sri Lanka, and India (Figures 1B,C; Peel et al., 2007). For each of the three species, up to five individuals from 4 to 8 nests were collected from more than five populations each. *S. sarasinorum* spiders were collected in October through December 2010 and transported at ambient temperature inside intact nests to the laboratory in Denmark, where they were frozen at −20°C. *S. dumicola* and *S. mimosarum* were collected in April through June 2012, cut in half and submerged in ATL buffer (from DNeasy Blood and Tissue kit, Qiagen, Hilden, Germany) in the field, and transported to the laboratory at ambient temperature where they were stored at −20°C. These samples were previously used for investigating population genetics of these species (Settepani et al., 2017). See Supplementary Table S1 for a sample summary and Supplementary Table S3 for a full sample list.

Spiders used for imaging were adult female *S. dumicola* collected in Namibia in June 2017 and transported to Denmark in intact nests.

### DNA Extraction and 16S rRNA Gene Amplicon Sequencing

DNA was extracted from whole spiders using DNeasy Blood and Tissue Kit (Qiagen) according to the manufacturer’s protocol. 16S rRNA gene amplicon libraries were prepared according to Illumina’s 16S Metagenomic Sequencing Library Preparation guide, with slight modifications, using Bac341F and Bac805R primers to amplify variable regions V3 and V4 (Herlemann et al., 2011). Sequencing was done in five runs on a MiSeq desktop sequencer (Illumina, San Diego, CA, United States). Some samples did not yield a PCR product after the first or second PCR steps and we therefore had to optimize PCR conditions for these samples individually by e.g., increasing PCR cycle number, using a different DNA polymerase, or including gel extraction (for details see Supplementary Methods, Supplementary Figure S1 where the affected samples are indicated, and Supplementary Figures S5–S9 showing all analyses based on data excluding the affected samples).

### Full-Length 16S rRNA Gene Sequencing

Six *S. dumicola* samples (M897, M538, X435, M898, M754, and M562) were selected to obtain full-length 16S rRNA gene sequences of dominant symbionts, for proper phylogenetic identification. The near full-length 16S rRNA gene was amplified using the general bacterial primers Eub26 F (Hicks et al., 1992) and 1492R (Loy et al., 2002). Clone libraries were prepared using the pGEM^®^-T Vector System (Promega, Madison, WI, United States) according to the manufacturer’s protocol. Sanger sequencing of clones was performed by GATC Biotech (Konstanz, Germany). Sequences were trimmed, assembled, and checked for chimeras using Geneious v11.0.5 (Biomatters Ltd., Auckland, New Zealand).

### 16S rRNA Gene Amplicon Analysis

All analyses were run on local servers using R v. 3.4.4 (R Core Team, 2018) and custom shell scripts. Raw sequences were trimmed to remove barcodes and PCR primers using cutadapt v1.18 (Martin, 2011). Filtering, denoising, paired-end merging, and classification was done using the R package “dada2” v. 1.6.0 (Callahan et al., 2016). Sequences from each of five sequencing runs were filtered and denoised separately, so the Divisive Amplicon Denoising Algorithm (DADA) could make independent error models for each run. After denoising, the five data sets were merged for chimera finding, and Amplicon Sequence Variants (ASVs) were classified using the Silva SSU reference database nr. 132 (Quast et al., 2013). The R package “phyloseq” v. 1.22.3 (Mcmurdie and Holmes, 2013) was used to extract data as separate ASV, taxonomy, and sample tables, ASVs were filtered to exclude non-bacteria and ASVs <400 bp. After excluding samples with <3000 filtered reads, 58 *S. dumicola* samples, 60 *S. mimosarum* samples, and 98 *S. sarasinorum* samples remained for further community analysis.

Normalization of amplicon data was done in one of two ways: For any analysis involving diversity measures, amplicon reads were subsampled to a common depth of 3000 reads. For everything else, amplicon reads are reported as fractions of all reads per sample. If subsampling was used, it is stated in the figure caption.

All remaining analyses and visualizations of community data were done with custom R scripts using several R packages such as “ggplot2” v. 3.0.0 (Wickham, 2009), “vegan” v. 2.5-6 (Oksanen et al., 2019), and “Biostrings” v. 2.52.0 (Pagès et al., 2019). Custom scripts and their use are listed in Supplementary Table S2; they are available in the GitHub repository https://github.com/Mettetron/3Species.

Read statistics, detailed sample information, raw and normalized ASV data, and microbiome taxonomic information including ASV sequences are available in the supporting information (Supplementary Tables S1, S3–S6).

### Phylogenetic Analysis

Phylogenetic trees based on full length 16S rRNA gene sequences were constructed using Bayesian inference. Sequences were aligned using MUSCLE (Edgar, 2004) via the R package “muscle” v. 3.26.0. Alignments were filtered to exclude columns with more than 50% gaps, and alignments were manually curated in ARB (Ludwig et al., 2004). Trees were constructed with MrBayes (Ronquist et al., 2012) using a generalized time reversible substitution model with a gamma distributed among-site rate variation and a proportion of invariable sites (GTR+I+Γ), the analysis was run with 1,000,000 generations and a sample frequency of 100. The shorter ASV sequences were added to the resulting consensus trees by maximum parsimony using the “add marked partial sequence” function in ARB without changing the overall tree topology.

### Design and Optimization of Probes for FISH

A new 16S rRNA-targeted oligonucleotide probe for FISH-detection of *Borrelia* spp. (Bor744: 5′-ACTCAGCGTCAGTCTTGA-3′) was developed using the probe design function in ARB (Ludwig et al., 2004). The probe matches all spider-associated *Borrelia*-like ASVs and full-length sequences found in this study, as well as 123 out of 129 published *Borrelia* and *Borreliella* 16S rRNA sequences in the Silva SSU Ref NR 99 release 128 database (Quast et al., 2013), with no exact matches outside of this group. The probe binds to *Escherichia coli* position 744–761, an area of low accessibility (Fuchs et al., 1998); thus four helper probes (H726SPB: 5′-CCTAGAAGTTCGCCTTCG-3′; H649SPB: 5′-TCCCCTATCAGACTCTAGCTT-3′; H576SPB: 5′-AAACCGCCTACTCACC-3′; H762BOR: 5′-CTCCCTACGCTTTCGTG-3′) were designed specifically for the target *Borrelia*-like ASV_3. Hybridization efficiency and optimal formamide concentration were modeled using MathFISH (Yilmaz et al., 2011) and experimental optimization with *Borreliella sinica* DSM23262 showed that probe Bor744 required 25% formamide in standard FISH buffer (Pernthaler et al., 2001) for specific hybridization. In addition, probe LGC0355b was used with 35% formamide to specifically detect *Mycoplasma* spp. (Neulinger et al., 2009), and probes EUB I-III (Daims et al., 1999) and NON (Manz et al., 1992) were used as positive and negative controls, respectively. All probes were synthesized by biomers.net (Ulm, Germany).

### Sample Preparation and FISH

Spiders were sedated with CO_2_ gas and dipped in 70% ethanol to reduce surface hydrophobicity. The prosoma and opisthosoma were cut apart, and the tissue was fixed in 4% paraformaldehyde for 24 h at 4°C. Embedding in Tissue Freezing Medium (Leica), cryosectioning, FISH, mounting in Citifluor/Vectashield, and microscopy were performed as previously described in detail (Kroer et al., 2016), with the following modification: Embedded and frozen samples were kept at −80°C for long term storage, moved to −20°C at least 24 h before sectioning, and spiders were cut into 20 μm thick transverse sections at −15°C. FISH was performed with mono-labeled probes; after FISH, the cover slips were sealed to the slides with nail polish, and slides were stored at −20°C until use. Confocal imaging was done on a Zeiss (Oberkochen, Germany) LSM700 confocal laser scanning microscope (CLSM) with 405, 488, and 639 nm lasers and ZEN software. All images were processed using FIJI (Schindelin et al., 2012).

### Data Availability

Full length 16S rRNA gene sequences were submitted to NCBI with the accession numbers MH627291-MH627379. The V3–V4 16S rRNA gene amplicon sequences were submitted to NCBI Sequence Read Archive (SRA) with the accession numbers SRP130746, SRP130740, and SRP130742. Processed 16S rRNA gene amplicon data are available in supporting information (Supplementary Tables S3–S6).

## Results

### Social *Stegodyphus* Carry Low Diversity Microbiomes, Which Are Dominated by Single but Varying ASVs

**FIGURE 2:**
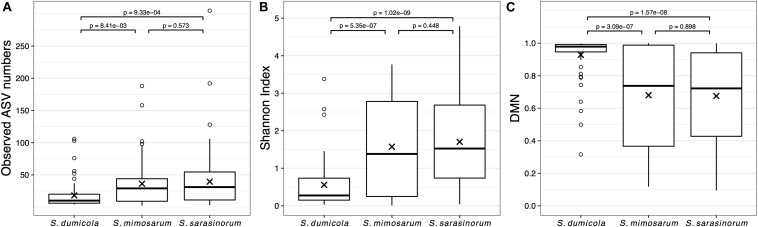
Microbiome richness, diversity, and dominance metrics for each of the three social *Stegodyphus* species based on subsampled data. Each data point is an individual spider. **(A)** Microbiome richness, represented by the number of observed ASVs. **(B)** Microbiome diversity represented by the Shannon index. **(C)** Microbiome dominance represented by McNaughton’s dominance (DMN) = sum of the relative abundances of the two most abundant ASVs in each sample. “x” indicates the means, *p*-values are based on pair-wise *t*-tests with Benjamini–Hochberg adjustment for multiple comparisons. *n* = 58 (*S. dumicola*), *n* = 60 (*S. mimosarum*), *n* = 98 (*S. sarasinorum*).

**FIGURE 3:**
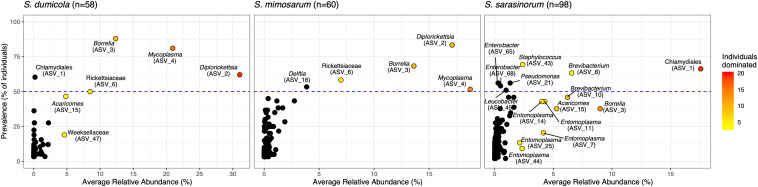
Prevalence and average relative abundance of ASVs in the three social *Stegodyphus* species (presence threshold: relative abundance ≥ 0.01%). Colored circles depict dominant ASVs; the color scale shows the number of animals in which a given ASV had a relative abundance of more than 30%. The horizontal dashed line indicates 50% prevalence; ASVs above the line are included in the core microbiome of the respective spider species.

**FIGURE 4:**
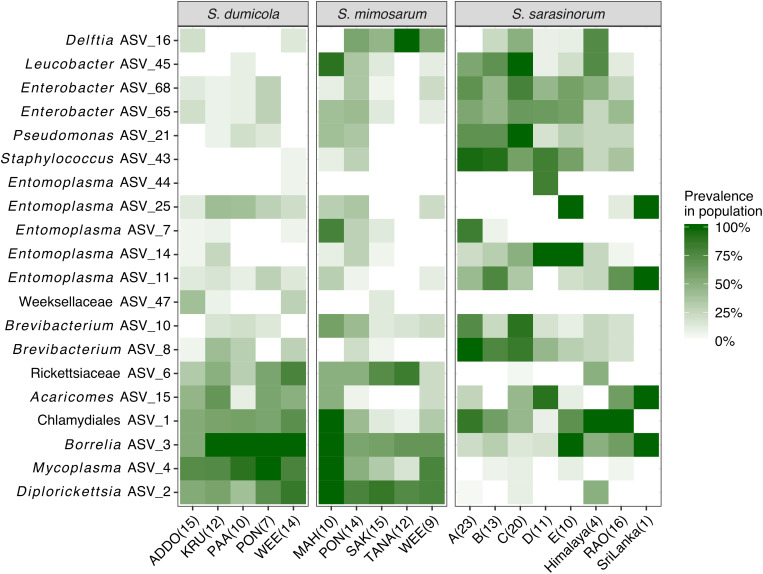
Heatmap of the core and dominant ASVs across the three social *Stegodyphus* species. Each column represents a population. The prevalence in a population is calculated as percent of individual spiders with a given ASV (presence threshold: Relative abundance ≥ 0.01%). The number of spider individuals in each population is shown in brackets.

Amplicon sequencing of 216 individual spiders from 85 nests revealed a total of 772, 1470, and 1567 bacterial 16S rRNA gene ASVs in *S. dumicola*, *S. mimosarum*, and *S. sarasinorum*, respectively (Supplementary Table S1). ASV richness (expressed as the number of observed ASVs per individual spider) was significantly lower in *S. dumicola* than in *S. mimosarum* and *S. sarasinorum* (Figure 2A) but with 24–47 ASVs overall fairly low. Likewise, ASV diversity (Shannon index) was significantly lower in individuals of *S. dumicola* than in the other two species (Figure 2B and Supplementary Table S1). The majority of all ASVs (83%) occurred in only 1–2 samples and at relative abundances <0.1% and thus can be categorized as transient microbiome. 65% of the individual spiders were dominated by a single ASV, accounting for more than 50% of the microbiome (Supplementary Figure S1). The McNaughton’s dominance index (DMN, the sum of the two most abundant ASVs, Figure 2C) shows that dominant ASVs are more common in *S. dumicola* than in *S. mimosarum* and *S. sarasinorum*, which matches the much lower alpha diversity in *S. dumicola*. Not all spiders were dominated by the same ASV but only a small set of 15 ASVs consistently showed a dominant role (Figure 3); this includes the most abundant and most prevalent ASVs (Figure 4), which were classified as Chlamydiales (ASV_1), *Diplorickettsia* (ASV_2), *Borrelia*, (ASV_3), *Mycoplasma* (ASV_4), and Rickettsiaceae (ASV_6). In addition, a few taxa with overall lower abundance and prevalence (*Rickettsia*, *Entomoplasma*, *Brevibacterium*, *Acaricomes*, *Staphylococcus*, Weeksellaceae) were occasionally dominant in *S. dumicola* or *S. sarasinorum* (Figure 3).

### The Three Social *Stegodyphus* Species Carry Distinct Microbiomes but No Ubiquitous Endosymbionts

Analysis of similarities (ANOSIM) based on Bray–Curtis dissimilarities revealed that the overall microbiome composition differed significantly (*p* < 0.01) between the three *Stegodyphus* species, but that it was more similar between the two African species, *S. dumicola* and *S. mimosarum* (*R* = 0.094), than between any of them and the Indian species, *S. sarasinorum* (*R* = 0.554 with *S. dumicola* and *R* = 0.499 with *S. mimosarum*).

The interspecies similarities and differences of the microbiomes were even more evident when looking at each host species’ core microbiome (here defined as ASVs present in >50% of the individuals with >0.01% relative abundance): The two African species, *S. dumicola* and *S. mimosarum*, each contained five core ASVs, of which four were shared: *Diplorickettsia* (ASV_2), *Borrelia* (ASV_3), *Mycoplasma* (ASV_4), and Rickettsiaceae (ASV_6). The fifth core ASV in *S. dumicola* was Chlamydiales (ASV_1), in *S. mimosarum* it was *Delftia* (ASV_16) (Figure 3). The core microbiome of the Indian species *S. sarasinorum* contained seven ASVs, of which only Chlamydiales (ASV_1) was shared with the *S. dumicola* core (Figure 3). No single ASV could be detected in all spider individuals, and the only ASV found in all nests of a species was *Diplorickettsia* (ASV_2) in *S. mimosarum*. However, all core ASVs were detected in all populations of their respective host species (Figure 4). Although not part of any of the three core microbiomes, *Acaricomes* (ASV_15) was also detected in all populations of *S. dumicola* and in most populations of *S. mimosarum* (3 out of 5) and *S. sarasinorum* (7 out of 8). ASV_3 (*Borrelia*) was overall most prevalent even though it was not part of the *S. sarasinorum* core microbiome (Figures 3, 4).

### Phylogenetic Analysis of Core and Dominant ASVs Indicates the Presence of *Stegodyphus*- and Arachnid-Specific Lineages

**FIGURE 5:**
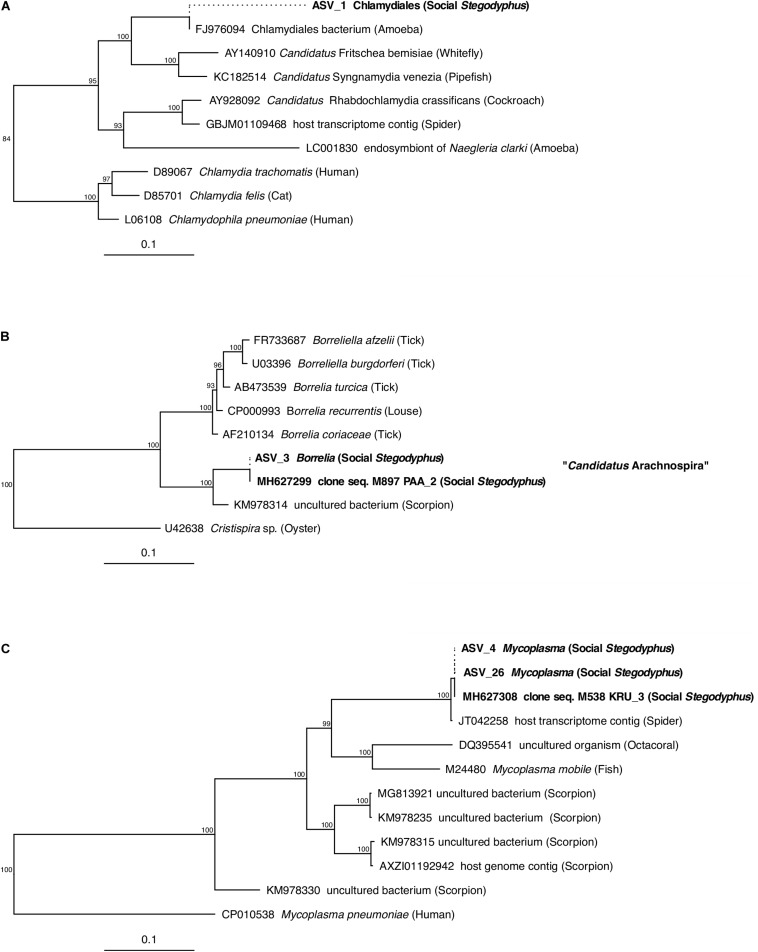
Phylogenetic position of three abundant, prevalent, and putatively specific *Stegodyphus* symbionts classified as **(A)** Chlamydiales, **(B)** *Borrelia* (*Candidatus* Arachnospira), and **(C)** *Mycoplasma*. Sequences obtained from *Stegodyphus* spiders are shown in bold font, host organisms are given in parenthesis. Trees are based on near full-length 16S rRNA gene sequences (identified by GenBank accession numbers) and calculated by Bayesian Inference. Short sequences (indicated by dotted lines, e.g., of ASVs) were added without changing the tree topology. Numbers on nodes are posterior probabilities. Scale bar: 0.1 estimated substitutions per site.

Of the core microbiome members, the ASVs classified as Chlamydiales (ASV_1), *Borrelia* (ASV_3), and *Mycoplasma* (ASV_4) were with 89, 97, and 91% only distantly related to their closest match in the database. Phylogenetic analysis based on partial (ASV_1) or full-length (ASV_3, ASV_4) 16S rRNA gene sequences identified each of these three putative symbionts as a novel lineage, hitherto only found in *Stegodyphus* (Figure 5). The phylogenetic placement of ASV_1 is tentative because of the short sequence available. It grouped with bacteria found in other animals, but seemingly share a more recent common ancestor with an amoeba-associated Chlamydiales bacterium (Figure 5A). The *Borrelia*-lineage formed a distinct monophyletic group with sequences obtained from scorpions, indicating that this lineage was arachnid-specific (Figure 5B), while the *Mycoplasma*-lineage grouped outside of the scorpion-associated *Mycoplasmas* (Figure 5C) although having higher full length sequence similarity with members of that group (Supplementary Table S8). The ASVs classified as *Diplorickettsia* (ASV_2) and Rickettsiaceae (ASV_6) were both >99% identical to sequences obtained from ticks, and clustered separately from non-arachnid hosts (Supplementary Figures S2A,B). The remaining ASVs of the core microbiome (*Delftia* in *S. mimosarum*; *Staphylococcus*, *Brevibacterium*, *Pseudomonas*, *Enterobacter*, and *Leucobacter* in *S. sarasinorum*; Figure 2) were all 100% identical to published sequences of diverse (non-arachnid) host-associated or environmental bacteria (Supplementary Table S8).

Besides members of the core microbiome, three other ASVs regularly dominated in individual spiders. ASV_15 grouped within the genus *Acaricomes* and showed 98% identity to *A. phytoseiuli*, isolated from a mite (Supplementary Figure S2C). ASV_12, classified as Weeksellaceae, was 97% identical to a shrimp gut-derived sequence (Supplementary Table S8) and formed a distinct cluster with two more ASVs within the *Bergeyella-Chryseobacterium* group (Supplementary Figure S2D). ASV_7 grouped with three other ASVs within the genus *Entomoplasma* and was 98% identical to *E. freundtii*, isolated from a beetle (Supplementary Figure S2E and Supplementary Table S8).

### Specific Symbionts Reside in a Complex Intestinal Tract

**FIGURE 6:**
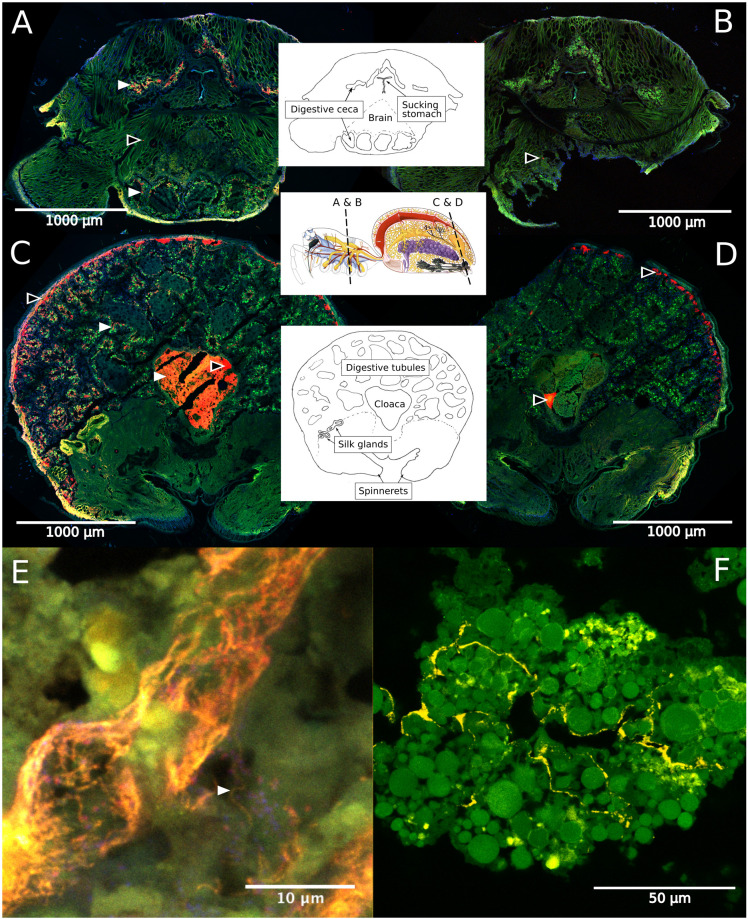
Fluorescence *in situ* hybridization (FISH) on cryosections of adult female *S. dumicola*. Middle panels show anatomy and location of sections. All panels: Green is autofluorescence, blue is DNA (DAPI) **(A,C)** Red is all bacteria (Eub I-III probe), white arrows point to examples of bacterial colonization. **(B,D)** No-hybridization controls (NON probe). **(A–D)** Black center arrows point to unspecific binding/autofluorescence. **(E)** Closeup of diverticula tissue. Orange is *Candidatus* Arachnospira hybridized with a specific probe (Bor477), white arrow pointing to clear spirochetal cell, red is all bacteria (Eub I-III probe). **(F)** Closeup of diverticula tissue. Yellow is *Mycoplasma* (LGC0355b probe).

Fluorescence *in situ* hybridization revealed the social spiders’ digestive system as the primary site for their microbiome. Spiders have a branching midgut, which fills most of the opisthosoma and even has lobes extending into the legs and head region of the prosoma (Foelix, 2011). The gut terminates in a rectal sac (the cloaca), where waste products are stored prior to excretion through the anus. In *S. dumicola*, the entire midgut, including the diverticula, was densely colonized with bacteria (as detected by the general bacterial probe EUB), which mostly lined the epithelial tissues and completely filled the cloaca (Figures 6A–D). FISH with probes specific for *Borrelia* and *Mycoplasma* (including the ASV sequences of this study) proved that these putative symbionts and members of the core microbiomes of *S. dumicola* and *S. mimosarum* lined the epithelium of the branching midgut, both of them interspersed with other bacteria (Figures 6E,F).

### Microbiomes Show Higher Similarity Within Nests Than Between Nests in All Three Host Species, and Interspecific Differences in Population Patterns

**FIGURE 7:**
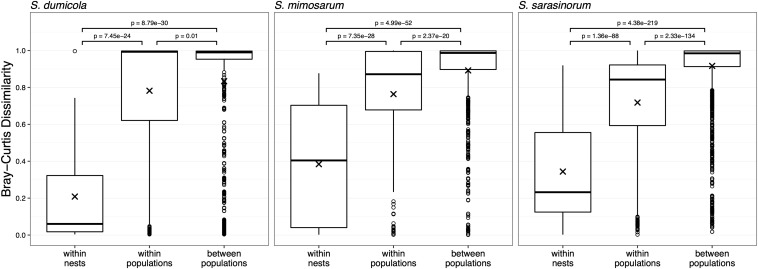
Beta diversity at three different host organization levels (within nest, within population, and between populations) in the three species of social *Stegodyphus*. Each data point compares the microbiomes of two individual spiders. Bray–Curtis dissimilarities were based on subsampled data. Group means are indicated with “x” and compared using one-way ANOVA and pairwise *t*-test with Benjamini–Hochberg adjusted *p*-values.

For each host species, we calculated microbiome beta diversity at three levels; within nests, within populations, and between populations (Figure 7). The microbiomes of spider individuals from the same nest were moderately to highly similar, with mean Bray–Curtis dissimilarities (BC) <0.4, and were with few exceptions dominated by the same ASV, although differences in relative abundance of the ASVs shared between individuals did sometimes occur (Supplementary Figure S1). Microbiomes within populations were more dissimilar (BC 0.71–0.78) but still significantly (*p* < 0.01) more similar than between populations (BC 0.83–0.92). This pattern was also evident when calculating the Sørensen dissimilarity index and UniFrac distance (Supplementary Figure S3), except that it was not significant for *S. dumicola*. Microbiome dissimilarity (expressed as BC dissimilarity) did not correlate with geographical distance for nests of *S. dumicola* and *S. mimosarum* (Supplementary Figures S4A,B). However, for *S. sarasinorum*, there was a weak but significant positive correlation between nest distance and microbiome dissimilarity (Supplementary Figure S4C).

**FIGURE 8:**
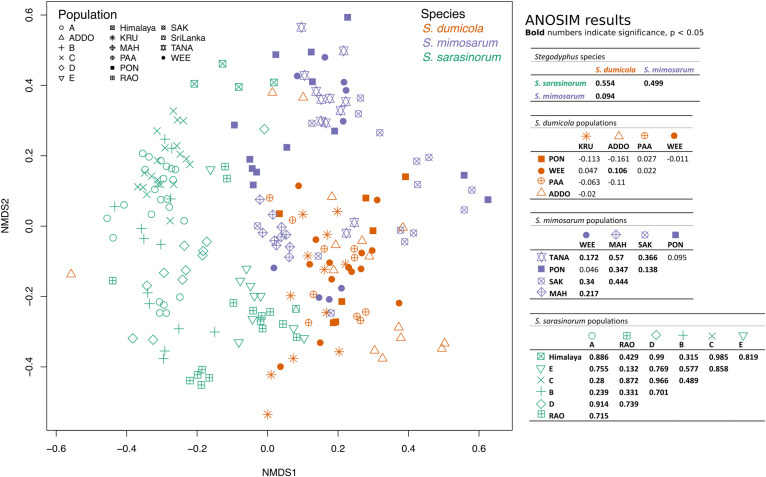
Non-metric multidimensional scaling (NMDS) ordination of spider microbiomes based on Bray–Curtis dissimilarities (with subsampled data). Each point is an individual spider. Orange: *S. dumicola*, purple; *S. mimosarum*, and green *S. sarasinorum*. Symbols denote the different populations. Stress = 0.23. The ANOSIM R metric indicates degree of difference between groups; *R* = 0 indicates no difference in microbiome composition between groups, *R* = 1 indicates a high degree of difference in microbiome composition between groups, and negative *R* values indicate more difference within a group than between groups. Bold numbers indicate significance (*p* < 0.05). The *S. sarasinorum* Sri Lanka population was removed from the ANOSIM analysis because it contains only one sample.

To test for host population-specific microbiome patterns, we conducted non-metric multidimensional scaling (NMDS) and ANOSIM analyses based on BC dissimilarities (Figure 8). Microbiomes grouped by host species as stated above but not consistently by host population: for *S. dumicola*, microbiomes were not significantly different between populations; in fact, the differences within a population were often larger than between populations (indicated by negative *R* values; Figure 8). For *S. mimosarum*, only the Madagascan populations MAH and SAK had microbiomes significantly different (*p* < 0.01) from each other and from the remaining three populations (*R* = 0.217–0.57). Finally, all populations of *S. sarasinorum* had distinct (*p* < 0.01) microbiomes (*R* = 0.24–0.99) except for populations E and RAO, whose microbiomes were indistinguishable (Figure 8).

## Discussion

### Social Spider Microbiomes Resemble Those of Social Insects and Other Arachnids in Structure but Contain Unique *Stegodyphus*-Specific Symbionts

The simple microbiome structure observed in the individuals of all three social *Stegodyphus* species, with low alpha diversity, strong dominance of 1–2 bacterial species (or ASVs), and a core microbiome of <10 species (Figures 2, 3, and Supplementary Figure S1), appears typical for the gut microbiomes of social arthropods (Corby-Harris et al., 2014; Sapountzis et al., 2015), with the notable exception of wood-feeding termites that rely on a complex microbiome for lignocellulose degradation (Benjamino and Graf, 2016). Solitary spider microbiomes are generally of low diversity (Zhang et al., 2018), and tend to be even more dominated by single symbionts than the social arthropods (Sheffer et al., 2020; White et al., 2020). With the current study design, we cannot deduce if the observed microbiome structure is specific for social *Stegodyphus* species or more general for the genus *Stegodyphus*.

Three of the most abundant and most dominant members of the social *Stegodyphus* core microbiomes define novel lineages indicative of specific endosymbionts: According to the 94.5% 16S rRNA similarity threshold proposed for bacterial genus delineation (Yarza et al., 2014), ASV_1 represents a novel genus within the obligate intracellular Chlamydiales (Kuo et al., 2015). Its tentative phylogenetic affiliation (Figure 5A) suggests it may reside inside spider-associated amoeba. ASV_3 is only distantly (92%) related to the obligate host-associated genus *Borrelia* (Barbour, 2018) and forms a novel genus together with a putative scorpion symbiont. Based on this phylogenetic information, a discriminant phenotype (spirochete morphology and inhabitant of the spider midgut including the diverticular tissue; Figure 6E), and its high prevalence in all three social *Stegodyphus* species (Figures 3, 4), we propose ASV_3 to represent a novel species within a novel genus, with the tentative name *Candidatus* Arachnospira stegodyphi (GenBank accession number for the full length 16S rRNA sequence: MH627299). Finally, ASV_4 groups with ASV_26 and a >99% identical sequence from the African sub-social spider *Stegodyphus tentoriicola* as a novel, presumably *Stegodyphus*-specific sister genus to the recently described Scorpion Mycoplasma Clade (Bolaños et al., 2019). These two clusters may delineate arachnid-specific evolutionary lineages of *Mycoplasma*, a typically host-associated bacterial group without cell wall and often intracellular infections (Brown et al., 2015); in the spider gut, they lined the gut epithelium and could either be located extracellularly or inside the apical border of the epithelium cells (Figure 6F).

Of the remaining core or dominant ASVs with specificity or preference for arachnid hosts, ASV_6 (*Rickettsiaceae*) and ASV_2 (*Diplorickettsia*) represent likely intracellular endosymbionts given their >99% identity to obligate intracellular symbionts of ticks, spiders, and insects (Mediannikov et al., 2010; Yu and Walker, 2015; White et al., 2020). In contrast, ASV_15 (*Acaricomes*) may represent an extracellular gut parasite similar to its close relative, *A. phytoseiuli*, which upon gut colonization in mites causes degradation of the epithelium (Schütte et al., 2008).

Most research into bacterial symbionts of spiders has focused on a set of bacteria that are sometimes involved in sex manipulation of insects and arachnids (Duron et al., 2008; Goodacre, 2011; Vanthournout et al., 2014). These bacteria, *Wolbachia*, *Rickettsia*, *Cardinium*, and *Spiroplasma*, were largely absent in social *Stegodyphus* (Supplementary Table S5). These symbionts probably do not play a role in the skewed sex ratio in social *Stegodyphus*, which has been explained by a biased production of X chromosome-carrying sperm cells (that give rise to females) in *S. dumicola* and *S. mimosarum* (Vanthournout et al., 2018).

### Individuals Within Nests Show Similar Microbiomes, but a Patchy Distribution of Dominant Symbionts Indicates That Random Processes Drive Microbiome Changes Among Nests

One of the proposed benefits of group living is access to and sharing of beneficial endosymbionts and gut microbes (Lombardo, 2008). In fact, social insects like ants, termites, bees, and bumblebees transmit and homogenize their gut microbiomes between colony members by fecal-oral transmission, trophallaxis, oral exchanges, and via the shared nest environment (Powell et al., 2014; Brune and Dietrich, 2015; Billiet et al., 2017; Zhukova et al., 2017). The high microbiome similarity between individuals from the same nest observed in the social spiders (Figure 7 and Supplementary Figure S1) may likewise be explained by continuous transmission and homogenization within the nest: Both (allo)maternal care and matriphagy (Junghanns et al., 2017, 2019) are potential routes of pseudo-vertical, exclusively maternal, transmission (as males die off before spiderlings emerge), while communal feeding (Schneider and Bilde, 2008) and the shared nest environment may lead to horizontal transfer and homogenization of the microbiome.

In contrast, the social spider microbiomes are very dissimilar between nests (Figure 7): Dominant ASVs often differ between neighboring nests (Supplementary Figure S1), and only a single ASV was detected in all nests of its *Stegodyphus* host species (ASV_ 2 *Diplorickettsia* in *S. mimosarum*, Figures 3, 4). This pattern of divergent microbiome composition among nests could indicate that no single symbiont is obligate for host survival and that the microbiome composition changes over time. Change in microbiome composition may happen during the establishment of new nests founded by single or a few mated females, where the absence of continuous buffering from a group of nest mates allows the colonization order of the symbionts to determine who will finally dominate the nest microbiome. Thereby, niche preemption, as hypothesized for the human microbiome (Litvak and Bäumler, 2019), may be an important factor in shaping the *Stegodyphus* microbiome. In that context, it is interesting that for *S. dumicola*, each nest tends to have only a single mitochondrial haplotype (indicating it was founded by a single matri-lineage), while within a population different haplotypes can be found (Johannesen et al., 2002), i.e., a similar pattern as seen in the dominating ASVs. This opens for the intriguing hypothesis that certain ASVs preferentially follow certain haplotypes when single, mated females establish new nests and microbiomes. However, unlike the microbiome composition, the mitochondrial haplotypes show a clear population pattern with the most similar mitochondrial DNA in neighboring nests, indicating a slower rate of change in mitochondrial DNA than in microbiome composition. This would be expected with pseudo-vertical transmission of bacterial symbionts via e.g., regurgitation feeding having a lower transmission fidelity than the direct inheritance of mitochondria.

Despite some apparent stochasticity during the microbiome assembly in new nests and populations, identical ASVs are found across the entire geographic range, from South Africa to Northern India, and across all three social spider species (Figures 1, 3, 4). How can these core microbes be maintained in the host species? We see two possible scenarios, which may apply to distinct subsets of the core microbes: (1) selection for a set of functionally essential symbionts, or (2) continuous transmission by environmental vectors.

The first scenario (most likely for the intracellular and most prevalent symbionts, like *Diplorickettsia* or *Candidatus* Arachnospira) implies that a given core ASV is present in (almost) every nest, but sometimes below our detection limit, and that the symbiont provides an essential function at least once during a nest’s lifespan. Only nests that maintain that symbiont will then proliferate; a fluctuating relative abundance of the symbiont (from nearly undetectable to dominant) may be driven by host life stage, prey type, or environmental conditions. Time series studies are needed to resolve whether such predicted fluctuations occur in the field.

The second scenario suggests that core microbes can be acquired from distant nests or environmental reservoirs. The spiders themselves are sit-and-wait predators, so unless the bacteria are airborne, they would need to be introduced to the nest by vectors, likely in the form of prey or predators. This appears plausible for non-host-restricted taxa like *Delftia*, *Pseudomonas*, or *Brevibacterium* (see Supplementary Table S8). The *Stegodyphus*- or arachnid-specific symbionts, however, would need a more specific vector. Migration of social spiders between nests is thought to be rare (Johannesen et al., 2002), but social and more migratory sub-social *Stegodyphus* species co-occur in both Africa and India (Settepani et al., 2017), and different social and sub-social species can be observed in the same nest (Trine Bilde, personal observation). Such mixing may suffice to transmit symbionts and maintain them in the host populations, and can potentially explain why the co-occurring African *S. dumicola* and *S. mimosarum* share almost all core ASVs, and generally have more similar microbiomes than they do with the Indian *S. sarasinorum* (Figures 3, 8), although *S. dumicola* and *S. sarasinorum* are genetically more closely related (Figure 1A; Settepani et al., 2016).

### Host Population-Specific Microbiomes Were Detected in *S. sarasinorum* but Not in the African Social Spiders

The microbiomes of all three spider species were more similar within a host population than between populations, based on significant differences in BC dissimilarity (Figure 7). These patterns could be explained by random processes of drift, or possibly some degree of local adaptation to the host population or to the environment. However, only for the Indian species *S. sarasinorum* and two of the Madagascan populations of *S. mimosarum*, the microbiomes clustered according to host populations (Figure 8), and only for *S. sarasinorum* there was a correlation between microbiome dissimilarity and distance between nests (Supplementary Figure S4). However, we acknowledge that, due to the compositional nature of amplicon-based data, even this weak correlation may be an overestimation (Gloor et al., 2017). The population structure of social *Stegodyphus* is driven by extinction-recolonization dynamics, which over time will reduce genetic variation among host populations (Settepani et al., 2017), and possibly also their associated microbiome. In addition, mixing of symbiont populations via migration, vectors, and environmental acquisition possibly occurs (see above). The extent of mixing and dispersal should then play a key role for the degree of homogenization (Settepani et al., 2014). Given the shorter geographic distances between populations and the greater number of co-existing social and sub-social *Stegodyphus* species (Settepani et al., 2017), it is conceivable that the African populations (except for the *S. mimosarum* “island” populations on Madagascar) experience a higher degree of microbiome homogenization than *S. sarasinorum* in India, and therefore do not show detectable microbiome population patterns.

Another factor may be the diversity of the microbiome or the number of core or dominating ASVs, which is lowest in *S. dumicola* and overall highest in *S. sarasinorum* (Figures 2–4): This may facilitate the detection of population-specific patterns at the 16S rRNA gene amplicon level in *S. sarasinorum*, while we cannot exclude that patterns may first occur at higher resolution (e.g., at the genome level) in the African species.

Correlating microbiome composition with population-level climate variation proved difficult because of the very weak population pattern in the microbiome data. Future studies might remedy this by measuring the microclimate of individual nests, or by doing transplant experiments.

## Conclusion and Perspectives

Our study revealed low-diversity microbiomes with a host species-specific pattern and novel symbiont lineages in social spiders. Most individual spider microbiomes were dominated by one out of 4–15 ASVs and were highly similar for spiders from the same nest, suggesting stable microbiome transmission within nests. In contrast, microbiomes varied strongly between nests, which points to the importance of niche preemption or other random factors during host dispersal and establishment of new nests. Based on the retention of specific symbionts across host populations and species in spite of their apparent low prevalence, we suggest a recurrent symbiont acquisition from environmental vectors and/or an essential contribution of the symbionts to the spider phenotype. Further studies need to test these hypotheses on transmission, temporal stability, and function of the symbionts. Population patterns in microbiome composition were too weak to single out specific symbionts that might play roles in local adaptation. However, stronger patterns may occur at higher genomic resolution, or with a focus on functional genes, and microbiome-mediated local adaptation cannot be excluded for the social spiders.

## Data Availability Statement

The datasets generated for this study can be found in the NCBI Sequence Read Archive SRP130746, SRP130740, SRP130742, NCBI GenBank MH627291–MH627379, and in the Supplementary Material.

## Author Contributions

MB, TB, and AS designed the research. VS and TB collected the field samples. MB, VS, JB, and ML performed the lab work. MB analyzed and visualized the data. MB, JB, ML, TB, and AS wrote and edited the manuscript. All authors contributed to the article and approved the submitted version.

## Conflict of Interest

The authors declare that the research was conducted in the absence of any commercial or financial relationships that could be construed as a potential conflict of interest.
