# Supplementary material for: Microbiomes and Specific Symbionts of Social Spiders: Compositional Patterns in Host Species, Populations, and Nests
Source: Front Microbiol. 2020 Jul 31;11:1845. doi: 10.3389/fmicb.2020.01845 (PMC7412444; doi:10.3389/fmicb.2020.01845)
Supplement: Supplementary file 1 [file Presentation_1.PDF]

Supplementary Material

1 Supplementary Methods

1.1 PCR protocol for amplicon run 0, 1, 2, and 3:

Three PCR amplifications were carried out for each sample, the first using Bac 341F and Bac 805R primers to amplify variable regions V3 and V4 (Herlemann et al., 2011), the second adding overhang adapters with modified primers and finally an index PCR with Nextera XT index primers (Illumina) for sample identification. Each PCR had a total reaction volume of 25 µl containing 2.5 µl DNA sample, 0.5 µl of each primer (primer stock concentration = 10pmol/µl), 12.5 µl 2x KAPA HiFi HotStart Ready Mix and 9 µl sterile H_2O. The PCRs were performed on a Veriti® 96-Well Thermal Cycler (Applied Biosystems®) with the following program: Denaturation at 95°C for 3 min. Cycles of 95°C, 55°C and 72°C for 30 secs each, and finally a 5 min extension step at 72°C. PCR 1 with 20 cycles, PCR 2 with 10 cycles and PCR 3 with 8 cycles. After each PCR, samples were purified using AMPure XP beads (Illumina).

1.2 PCR protocol for amplicon run 4:

Run 4 was a trouble shooting run, redoing PCRs and sequencing for samples which had failed to provide adequate read numbers in previous runs. Based on several trials and optimization iterations, the PCR protocol for samples in amplicon run 4 was carried out differently for different groups of samples. Table S7 summarizes these differences while the text below explains the details.

For several samples PCR 1 and PCR 2 were carried out normally as described for amplicon run 0-3, But some samples went through the following optimized protocol: PCR 1 was run with a diluted template and added BSA giving a total reaction volume of 25 µl containing 2.5 µl DNA sample (diluted 1:50), 0.5 µl of each primer (primer stock concentration = 10pmol/µl), 0.5 µm BSA (10mg/ml), 12.5 µl 2x KAPA HiFi HotStart Ready Mix and 8.5 µl sterile H_2O. This was run for 30 cycles rather than the 20 cycles normally run for PCR1. PCR 2 was run in triplicate for each sample with 3 µl DNA sample, 0.5 µl of each primer (primer stock concentration = 10pmol/µl), 12.5 µl 2x KAPA HiFi HotStart Ready Mix and 8.5 µl sterile H_2O, and for 12 cycles instead of the normal 10.

For all samples in amplicon run 4, PCR 2 products were size separated by agarose gel electrophoresis and the desired band was extracted from the gels using an E.Z.N.A. Gel Extraction Kit (Omega Bio-Tek). Several samples (see Table S7) in run 4 had such low DNA concentration after gel extraction that AMPure XP beads (Illumina) were used to bind and resuspend in a smaller volume. PCR 3 adding index primers was run as described for amplicon run 0-3, but with differing amounts of template depending on DNA concentration (see Table S7).

Herlemann, D. P., Labrenz, M., Jürgens, K., Bertilsson, S., Waniek, J. J., & Andersson, A. F. (2011). Transitions in bacterial communities along the 2000 km salinity gradient of the Baltic Sea. *The ISME Journal*, *5*(10), 1571–1579. doi: 10.1038/ismej.2011.41

2 Supplementary Figures and Tables

Table S1 Summary statistics of samples, sequencing, and community data

	S. dumicola	*S. mimosarum*	*S. sarasinorum*
**Samples**	58	60	98
**Nests**	28	23	34
**Populations**	5	5	8
**Raw read number[a,c]**	43079 (5278-675594)	51551 (14601-96639)	44425 (3959-280637)
**Filtered read number[a,d]**	30270 (3131-438346)	30368 (4059-68412)	31146 (3004-195228)
**ASV number[d]**	772	1470	1567
**ASV number per sample[a,d]**	24.52 (4-152)	44.73 (3-211)	46.97 (3-343)
**Shannon diversity[b,d]**	0.56 ± 0.67	1.58 ± 1.30	1.71 ± 1.11
**Simpson diversity[b,d]**	0.77 ± 0.25	0.48 ± 0.38	0.40 ± 0.29
**DMN[b,d]**	0.93 ± 0.13	0.68 ± 0.32	0.68 ± 0.27

Note: Based on samples with minimum 3000 reads after filtering
a: Mean (minimum-maximum)
b: Mean ± standard deviation
c: Unfiltered forward read numbers form Miseq
d: After quality filtering, denoising, paired-end merging, chimera finding, length filtering (minimum 400bp) and taxonomic filtering

**Table S2** R scripts used for analysis and data visualization		
**Use**	**Figure**	**GitHub link**
**Basic DADA2 analysis**	-	https://github.com/Mettetron/3Species/blob/master/DADA2_basicAnalysis.R
**Merging of ASV data from several sequencing runs**	-	https://github.com/Mettetron/3Species/blob/master/DADA2_combRuns_tax.R
**Data formatting**	-	https://github.com/Mettetron/3Species/blob/master/DADA2_to_Phyloseq.R
**Decontamination and normalization**	-	https://github.com/Mettetron/3Species/blob/master/DADA2_filterAndNorm.R
**Richness, alpha diversity and dominance boxplots**	2	https://github.com/Mettetron/3Species/blob/master/ASV_divDom_box.R
**Prevalence vs. abundance scatter plots**	3	https://github.com/Mettetron/3Species/blob/master/ASV_coreDom_scatter.R
**Prevalence heatmap**	4	https://github.com/Mettetron/3Species/blob/master/ASV_prevalenceHeatmap.R
**Diversity boxplots**	7	https://github.com/Mettetron/3Species/blob/master/ASV_Bdiv_boxplots.R
**Ordination**	8	https://github.com/Mettetron/3Species/blob/master/ASV_NMDS_ordination.R
**Stacked barchart for individual hosts**	S1	https://github.com/Mettetron/3Species/blob/master/ASV_barchartsIndividuals_dumicola.R
**Diversity vs. Geographical distance**	S4	https://github.com/Mettetron/3Species/blob/master/ASV_betaDist_vs_geoDist.R

Note: Example data for running scripts also available on GitHub https://github.com/Mettetron/3Species

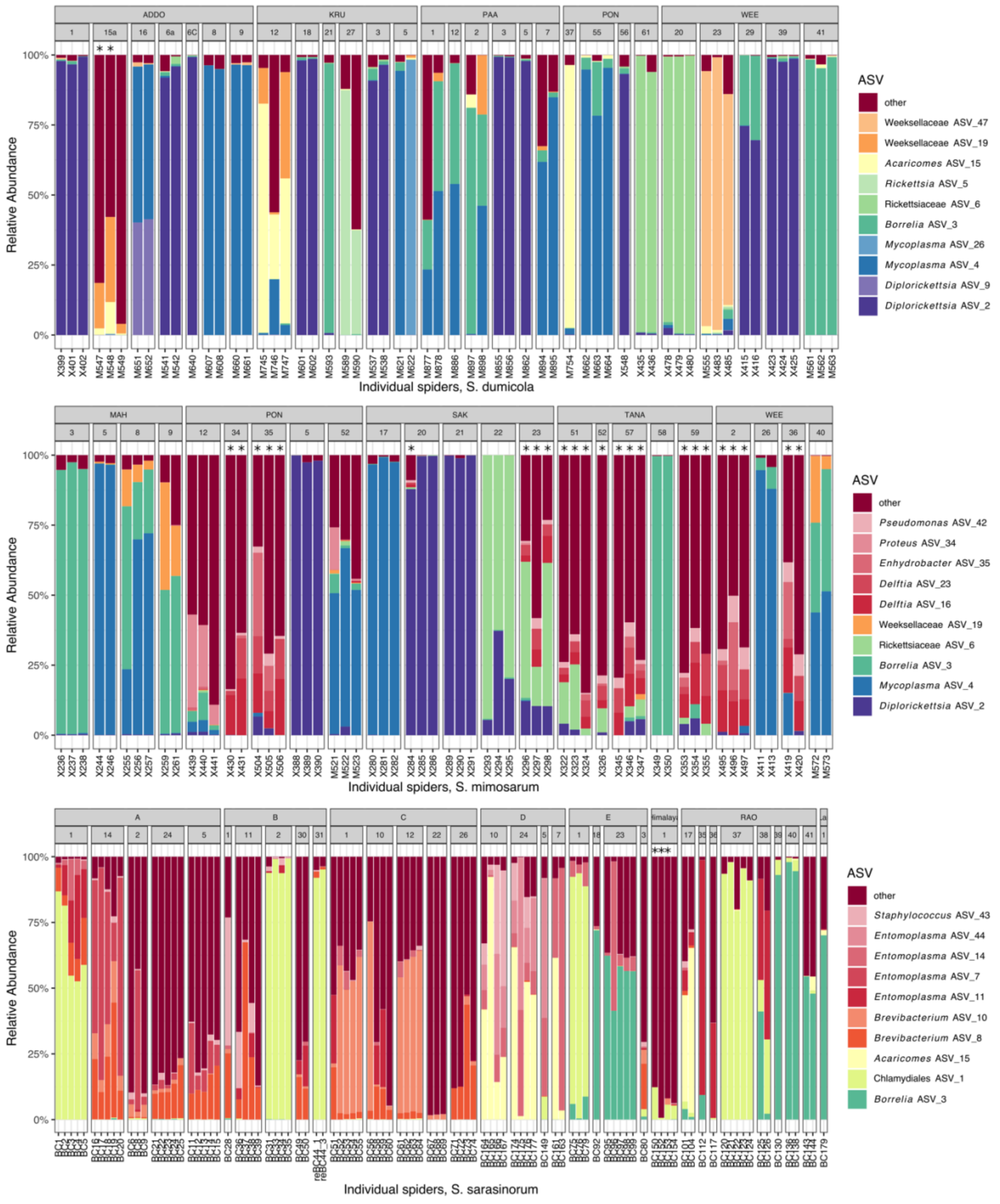

Supplementary Figure S1. Stacked bar charts showing relative abundances of the most abundant bacterial symbiont ASVs in individuals belonging to 3 species of social *Stegodyphus*. Each chart shows the 10 most abundant ASVs found in each spider species. The columns are grouped by spider population (top bar) and nest (bottom bar, numbers). Samples that required protocol optimization to yield 16S rRNA gene amplicons are marked with * above column.

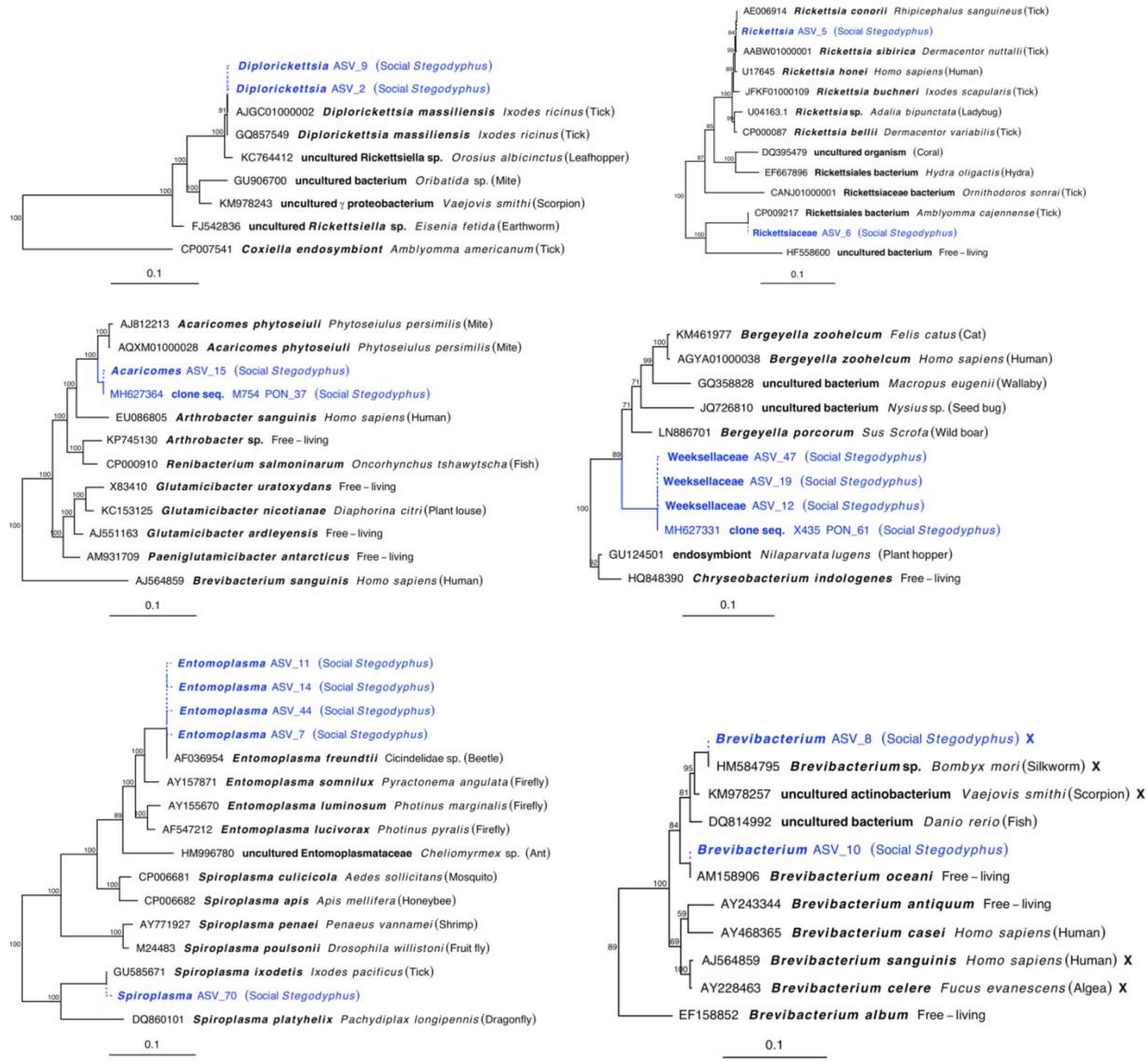

Supplementary Figure S2. Phylogenetic trees with dominant and core *Stegodyphus* symbionts. (A) *Diplorickettsia*, (B) *Rickettsia*/Rickettsiaceae, (C) *Acaricomes*, (D) Weeksellaceae, (E) *Entomoplasma*/*Spiroplasma*/*Mycoplasma*, (F) *Brevibacterium*. Bayesian inference consensus trees based on full length 16S rRNA sequences from bacteria closely related to social *Stegodyphus* symbionts found in the silva database (v128). Numbers on nodes show posterior probabilities. Blue text indicates sequences from this study. Short sequences (indicated by dotted lines, e.g. of ASVs) were added without changing the tree topology. Tip labels: Accession number, **full name**, and (host) of bacteria. Scalebar: 0.1 estimated substitutions per site. The placement of Brevibacterium ASV_8 was ambiguous and could have been placed with all tips marked with X.

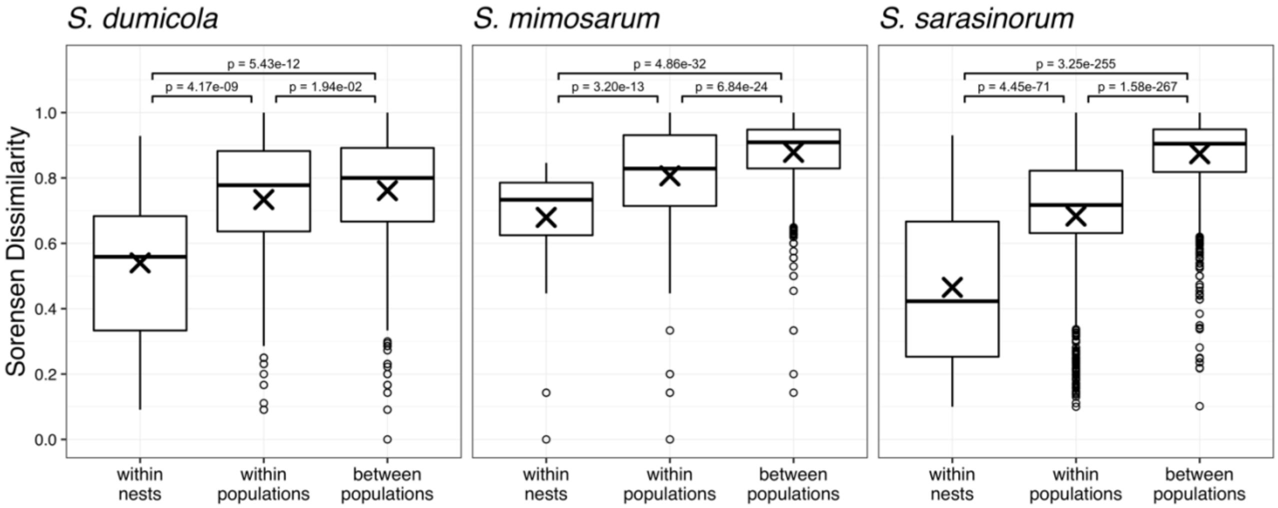

Supplementary Figure S3. Beta diversity at three different host organization levels (within nest, within population, and between populations) in the three species of social *Stegodyphus*. Each data point compares the microbiomes of two individual spiders. For estimating the Sørensen dissimilarities the data were subsampled to 3000 ASVs per spider. Group means are indicated with "x" and compared using one-way ANOVA and pairwise t-test with Benjamini-Hochberg adjusted p-values.

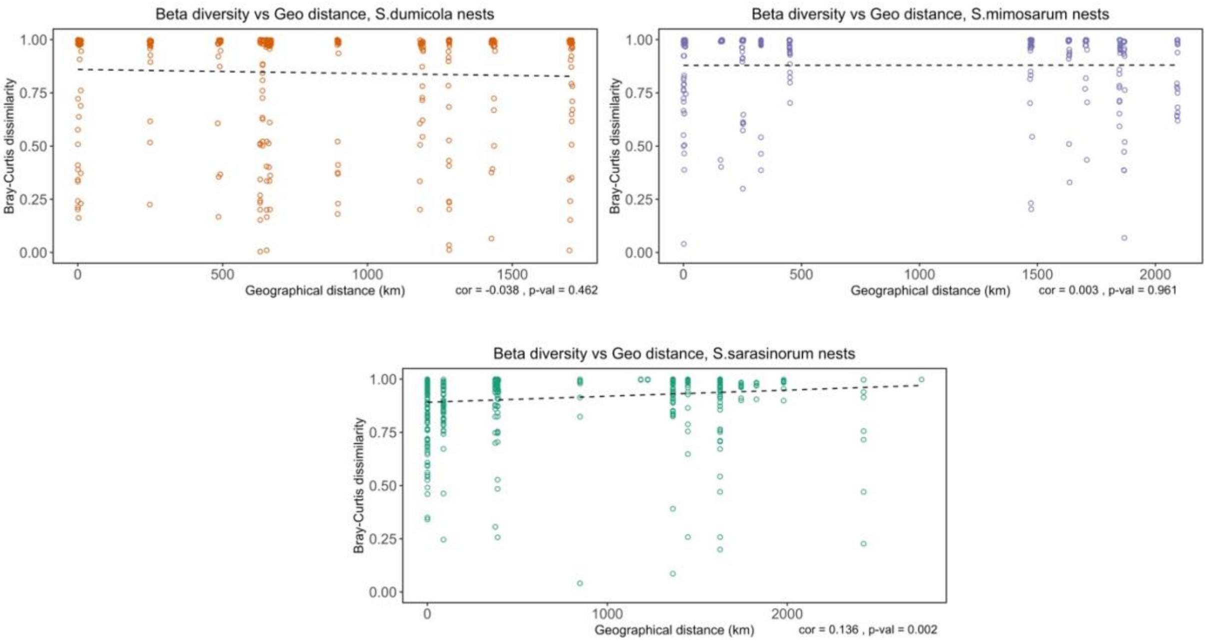

Supplementary Figure S4. Correlation of beta diversity and geographical distance between social *Stegodyphus* nests. Each data point represents comparison between average microbiomes of two nests. Beta diversity measures based on subsampled ASV data. Pearson's product-moment correlation.

Table S8 – ASV BLAST table

ASVs	Best BLAST match in the nucleotide collection database<br>Seq. ID[1]	Name	Source/Host	Accession
**ASV_1 Chlamydiales**	89% (381/428)	Uncultured *Chlamydia* sp.	Soil (Scotland)	LN831101.1
**ASV_2 *Diplorickettsia***	99% (426/427)	Uncultured *Diplorickettsia* sp.	Tick (*Ixodes scapularis*)	MN192053.1
**ASV_3 *Borrelia***	97% (414/427)	Uncultured bacterium clone	Scorpion (*Centruroides limpidus*)	KM978314.1
**Clone seq. MH627299**	95% (1426/1500)	Uncultured bacterium clone	Scorpion (*Centruroides limpidus*)	KM978314.1
**ASV_4 *Mycoplasma***	91% (389/426)	Uncultured bacterium clone	Plant (*Tamarix nilotica*)	JF302694.1
**Clone seq. MH627308**	86% (1276/1481)	Uncultured bacterium clone	Scorpion (*Vaejovis smithi*)	MG813921.1
**ASV_5 *Rickettsia***	100% (402/402)	*Candidatus* Rickettsia tarasevichiae	Tick (*Ixodes persulcatus*)	MN446745.1
**ASV_6 Rickettsiaceae**	99% (398/402)	Rickettsiales bacterium Ac37b	Tick (*Amblyomma cajennense*)	CP009217.2
**ASV_7 *Entomoplasma***	98% (417/427)	*Entomoplasma freundtii*	Beetle (Cicindelidae)	CP024962.1
**ASV_11 *Entomoplasma***	98% (418/427)	*Entomoplasma freundtii*	Beetle (Cicindelidae)	CP024962.1
**ASV_14 *Entomoplasma***	97% (416/427)	*Entomoplasma freundtii*	Beetle (Cicindelidae)	CP024962.1
**ASV_25 *Entomoplasma***	97% (416/427)	*Entomoplasma somnilux*	Beetle (*Pyractomena angulata*)	CP024965.1
**ASV_44 Entomoplasma**	97% (415/427)	*Entomoplasma freundtii*	Beetle (Cicindelidae)	CP024962.1
**ASV_8, ASV_10 *Brevibacterium***	100% (407/407)	100+ sequences. E.g. *B. sediminis*, *B. epidermidis*, *B. sanguinis*	E.g. Silkworm, Cell phone, Deep-sea sediment, Human	NA
**ASV_12 Weeksellaceae**	97% (408/422)	Uncultured bacterium clone	Shrimp (*Penaeus vannamei*)	KP953663.1
**Clone seq. MH627331**	95% (1416/1487)	*Bergeyella zoohelcum*	Cat (*Felis catus*)	KM461977
**ASV_47 Weeksellaceae**	96% (407/422)	Uncultured bacterium clone	Shrimp (*Penaeus vannamei*)	KP953663.1
**ASV_15 *Acaricomes***	99% (413/416)	*Acaricomes phytoseiuli*	Plant (*Vitis vinifera*) / Mite?	HM803939.1
**Clone seq. MH627364**	98% (1454/1483)	*Acaricomes phytoseiuli*	Mite (*Phytoseiulus persimilis*)	NR_042334
**ASV_16 *Delftia***	100% (427/427)	100+ sequences. E.g. *D. Tsuruhatensis*, *D. lacustris*, *D. siamensis*	E.g. Bollworm, Mosquito larvae, Louse, Fresh water, Activated sludge,	NA
**ASV_21 *Pseudomonas***	100% (427/427)	100+ sequences. E.g. *P. parafulva*, *P. fulva*, *P. punonensis*, *P. straminea*	E.g. Moth larvae, Soil, Marine algae, Rice paddy, Drosophila	NA
**ASV_43 *Staphylococcus***	100% (427/427)	100+ sequences. E.g. *S. sciuri*, *S. aureus*	E.g. Cow, Monitor lizard, Human, Chili, Fish, Banana, Bollworm	NA
**ASV_45 *Leucobacter***	100% (409/409)	29 sequences. E.g. *L. tardus*	E.g. Sea cucumber, Fish, Citrus tree, Moth, Human	NA
**ASV_65 *Enterobacter***	100% (427/427)	95 sequences. E.g. *E. asburiae*, *E. tabaci*, *E. cloacae*, *E. hormaechei*	E.g. Potato root, Moth larve, Fish, Honey bee, River water	NA
**ASV_68 *Enterobacter***	100% (427/427)	100+ sequences. E.g. *E. bugandensis*, *E. hormaechei*, *Klebsiella pneumoniae*	E.g. Soil, Rice paddy, Mosquito pupae, Bee	NA

[1] Percentage match (identical bases/alignment length)

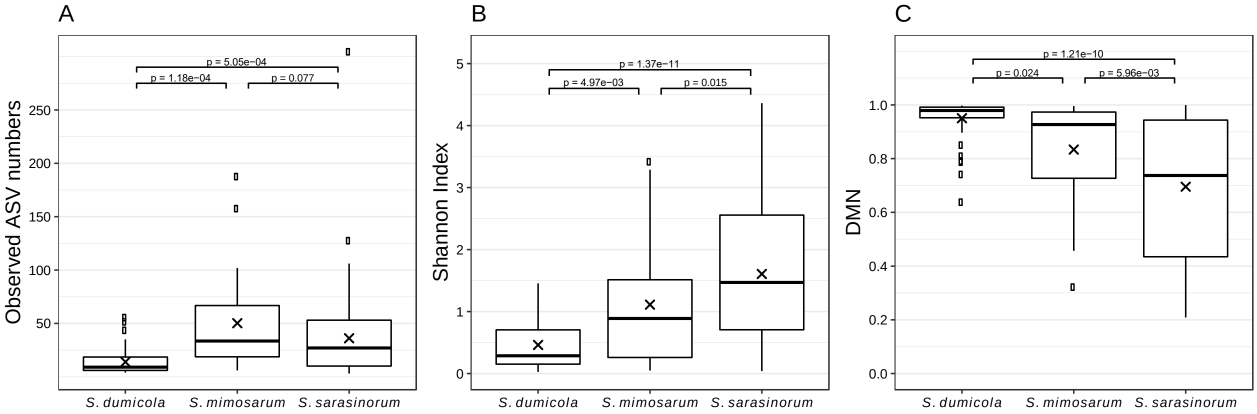

Supplementary Figure S5. Same as figure 2 in main text, but excluding all samples from sequencing run 4 (re-run and optimized samples).

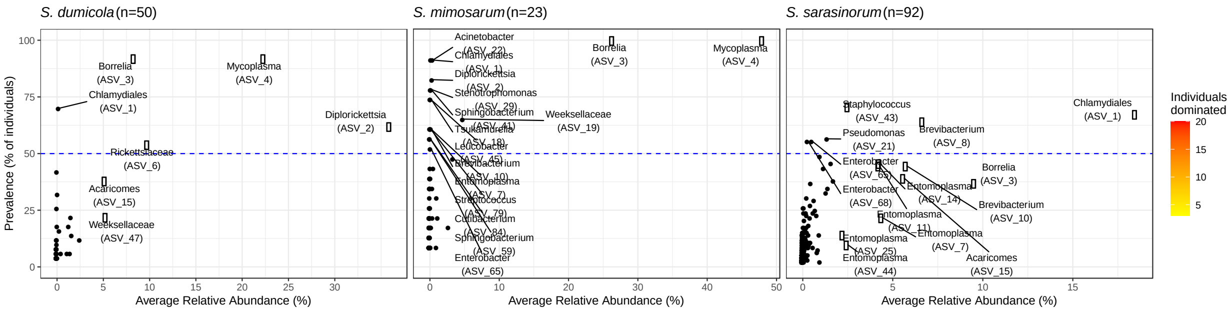

Supplementary Figure S6. Same as figure 3 in main text, but excluding all samples from sequencing run 4 (re-run and optimized samples).

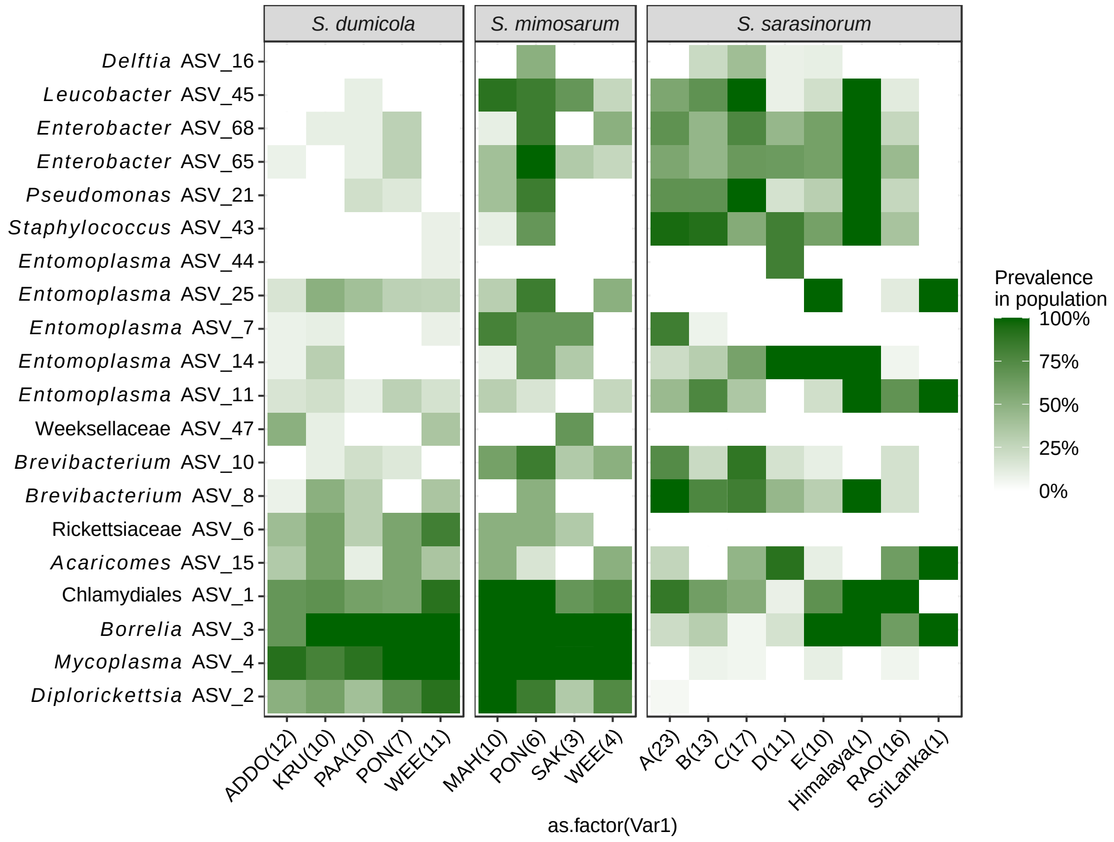

Supplementary Figure S7. Same as figure 4 in main text, but excluding all samples from sequencing run 4 (re-run and optimized samples).

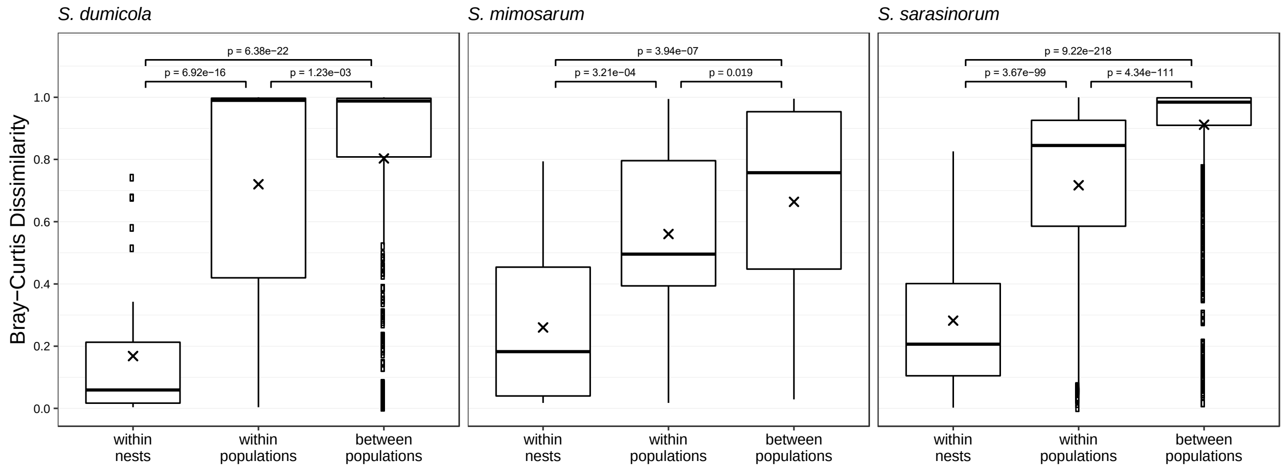

Supplementary Figure S8. Same as figure 7 in main text, but excluding all samples from sequencing run 4 (re-run and optimized samples).

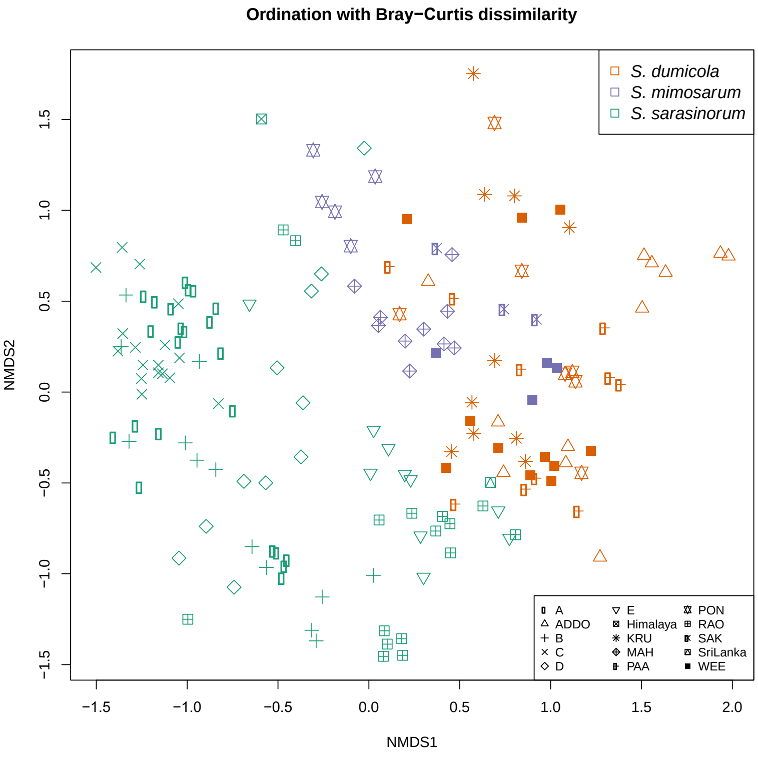

species		
	dumicola	mimosarum
sarasinorum	**0.569**	**0.315**
mimosarum	−0.009	

S. Dumicola populations				
	KRU	ADDO	PAA	WEE
PON	-0.104	0.028	0.027	0.013
WEE	0.042	0.108	0.089	0
PAA	-0.035	0.017	0	0
ADDO	0.109	0	0	0

S. Mimosarum populations			
	WEE	MAH	SAK
PON	0.234	**0.394**	0.315
SAK	0.333	0.22	0
MAH	-0.047	0	0

S. Sarasinorum populations					
	A	RAO	D	B	C
E	**0.755**	0.132	**0.769**	**0.577**	**0.848**
C	**0.273**	**0.869**	**0.956**	**0.458**	0
B	**0.239**	**0.331**	**0.701**	0	0
D	**0.914**	**0.739**	0	0	0
RAO	**0.715**	0	0	0	0

Supplementary Figure S9. Same as figure 8 in main text, but excluding all samples from sequencing run 4 (re-run and optimized samples).
